# The Effects of Crocodile Blood Supplementation on Delayed-Onset Muscle Soreness

**DOI:** 10.3390/nu13072312

**Published:** 2021-07-05

**Authors:** Chirawat Paratthakonkun, Vipu Vimuttipong, Alisa Nana, Kornkit Chaijenkij, Ngamphol Soonthornworasiri, Dumrongkiet Arthan

**Affiliations:** 1 College of Sports Science and Technology, Mahidol University, Nakhon Pathom 73170, Thailand; vipu0120175@gmail.com (V.V.); alisa.nan@mahidol.ac.th (A.N.); kornkit.cha@mahidol.edu (K.C.); 2 Department of Tropical Hygiene, Faculty of Tropical Medicine, Mahidol University, Bangkok 10400, Thailand; ngamphol.soo@mahidol.ac.th; 3 Department of Tropical Nutrition and Food Science, Faculty of Tropical Medicine, Mahidol University, Bangkok 10400, Thailand

**Keywords:** DOMS, eccentric exercise, crocodile blood

## Abstract

Delayed-onset muscle soreness (DOMS) is associated with increases in acute inflammatory and biochemical markers, muscle swelling, pain, and reduced functional performance. This study aimed to investigate the preventative effects of crocodile blood supplementation on DOMS induced by eccentric exercise. Sixteen healthy males were randomly allocated to either a crocodile blood (CB, *n* = 8) or a placebo (PL, *n* = 8) treatment. Participants receiving the CB treatment consumed four capsules of freeze–dried CB powder (1 g day^−1^) over 18 days. Participants receiving the other treatment were administered a placebo over the same period. An eccentric exercise protocol was performed, and functional performance, visual analogue scale (VAS)-measured pain, knee range of movement (ROM), thigh circumference (swelling), and cytokines, enzymes, and biochemical parameters were assessed immediately after exercise as well as after 24 h, 48 h, and 72 h. CB supplementation could significantly maintain maximum voluntary isometric contraction (MVIC) at 24 h (*p* = 0.001) and 48 h after exercise (*p* = 0.001) when comparing values at different times for the CB group. In the CB group, thigh circumference decreased only immediately after eccentric exercise (*p* = 0.031) in comparison with pre-eccentric exercise values. An 18-day supplementation (1 g day^−1^) of crocodile blood does aid in the maintenance of functional performance and muscle swelling after eccentric exercise. Our data indicate that 1 g day^−1^ of crocodile blood supplementation should be safe for human consumption.

## 1. Introduction

High-intensity or unaccustomed exercise can lead to delayed-onset muscle soreness (DOMS), resulting in increased muscle stiffness, inflammation, swelling, and a prolonged reduction in muscle strength and exercise performance [1,2,3,4]. The functional loss after eccentric exercise is well documented to be an indirect marker of muscle soreness and is associated with elevated muscle cell-released enzymes, such as creatine kinase (CK) and lactate dehydrogenase (LDH) [5].

Muscle cell-released cytokines, including interleulin-6 (IL-6) and tumor necrosis factor-α (TNF-α), are widely used as inflammatory response indicators. Several research groups have shown IL-6, TNF-α, CK, and LDH levels to increase and peak 24 h after eccentric exercise [1,4,5,6].

The Thailand food and drug administration (FDA), has approved that crocodile blood (CB) products shall not exceed the maximum allowance level of 1 g per day as a dietary supplementation for human consumption [7]. Crocodile blood components have been reported to have antibacterial, antioxidant, and anti-inflammatory properties, confirming the high potential of crocodile blood as a dietary supplement [8]. Indeed, in a murine macrophage RAW 264.7 cell model, crude leukocyte extracts obtained from crocodile blood were found to have anti-inflammatory effects by reducing NO and IL-6, without impacting on TNF-α production [9]. Similarly, the anti-inflammatory activity of crocodile blood extracts was examined by measuring proinflammatory mediators in lipopolysaccharides-stimulated RAW 264.7 cells. Both crude leukocyte extracts and plasma can reduce nitric oxide production in a dose-dependent manner, but serum and hemoglobin cannot. In addition, this study confirmed the absence of cytotoxicity for the serum, plasma hemoglobin, and crude leukocyte extracts against RAW 264.7 cells [10]. The Thailand food and drug administration (FDA), has approved that crocodile blood (CB) products shall not exceed the maximum allowance level of 1 g per day as a dietary supplementation for human consumption [7]. In a mouse model, 7 days of whole blood and crocodile hemoglobin supplementations exhibited anti-inflammatory activity in both acute and chronic stages by significantly decreasing lipid peroxidation and reducing pro-inflammatory cytokine levels, e.g., IL-6, IL-1β, and TNF-α levels. In addition, a whole-blood sample and crocodile hemoglobin was found to improve cell viability of H_2_O_2_-induced oxidative damage in human fibroblasts, which may be attributed to the neutralization of free radicals [10]. Crocodile hemoglobin and its acid hydrolysates exhibited antioxidant activity and in vivo wound healing activity [11]. The antioxidant and anti-inflammatory peptides were extracted from hemoglobin hydrolysates of Siamese crocodile (*Crocodylus siamensis*) by utilizing HCl in an experiment simulating human stomach digestion. These processes were completed by pepsin digestion, and 2H hydrolysis (2HCHH) had the greatest anti-inflammatory efficacy in terms of reducing nitric oxide (NO) generation and dramatically reducing pro-inflammatory cytokines and inflammatory mediators, e.g.,interleukin-6 (IL-6), interleukin-1 beta (IL-1β), and prostaglandin-E2 (PGE2) production; 6-H hydrolysis (6H-CHH) showed the strongest antioxidant activity against nitric oxide radicals [12]. After feeding rats with iron deficiency anemia (IDA) with freeze–dried crocodile blood for at least four weeks, the hemoglobin levels of both normal and IDA rats significantly improved [13]. The composition of crocodile blood has been reported [7]. Crocodile blood supplementation contains large amounts of proteins (83.1%), and the remaining consists of essential vitamins and minerals, particularly iron [7]. Concerning the safety of the crocodile blood supplement, the serum, plasma hemoglobin, and crude leukocyte extracts exhibited no cytotoxicity in RAW 264.7 cells [10]. In a rat model, toxicity studies at acute, sub-chronic, and chronic levels of a freeze–dried crocodile blood product were carried out. Oral administration of 5000 mg/kg (body weight) per day or a dosage of six capsules/day for six months exhibited no toxicity in experimental rats. The results showed no change of hematological and biochemical parameters in rat serum, such as blood sugar, blood urea nitrogen (BUN), creatinine, alkaline phosphatase, and alanine transaminase. No adverse effect of crocodile blood products on liver and kidney functions was found. Thus, crocodile blood should be safe for consumption as a food supplement [7]. It is believed that crocodile blood holds the key for the maintenance of health because of its high anti-inflammatory activity that may reduce inflammation in human organs including muscle. Blood crocodile supplementation of 100 g contained 164 mg of iron [7]. A study in rats found that freeze–dried crocodile blood can effectively increase the hemoglobin and hematocrit levels of normal and IDA in rats [13]. Interestingly, there is evidence that iron supplementation in iron-deficient nonanemic endurance athletes can improve their iron status and aerobic capacity [14].

The aim of this study was to investigate the effects of CB supplementation on the inflammatory, biochemical, and functional performance responses to muscle-damaging exercise and to evaluate the short-term safety of CB in healthy males. It was hypothesized that CB would improve recovery of functional and inflammatory measures compared with a placebo and result in no adverse biochemical effects.

## 2. Materials and Methods

### 2.1. Participants

A total of 36 healthy sedentary males who exercised less than 150 min per week were recruited to participate in this study. Following withdrawals (*n* = 20; inability to attend all visits, injury or illness outside of trials, or inability to complete eccentric exercise protocol) 20 participants were excluded. The remaining 16 male participants completed the protocol (age, 20.9 ± 1.5 years; height, 173.14 ± 5.11 m; body mass, 65.22 ± 6.42 kg; body mass index (BMI), 21.72 ± 1.90 kg/m^2^; body fat, 16.43 ± 5.52%). The participants were free from cardiorespiratory and metabolic disease and were not currently taking any pharmacological agents or supplementations.

The participants were familiarized with all experimental procedures and associated risks and provided their written informed consent to participate. The study was approved by the Central Institutional Review Board, Mahidol University: MU-CIRB 2018/197.0810.

### 2.2. Crocodile Blood Preparation

CB supplementation was produced from *Crocodylus siamensis* by Sriracha Moda Co. Ltd., Chonburi, Thailand, under Good Manufacturing Practice (GMP) and Hazard Analysis Critical Control Point (HACCP) certification scheme for processing and safety management. The crocodile farming of Sriracha Moda Co. Ltd. was reviewed and approved to be the best management practice for crocodilian farming by Manolis et al. [15]. Healthy crocodiles aged approximately 3 years, both males and females, were chosen for CB production processes, including blood sample collection under antiseptic conditions, pasteurization, and lyophilization; freeze–dried CB was introduced in capsules. CB has been approved to be a safe supplementation for humans by the Food and Drug Administration Thailand (FDA).

### 2.3. Study Design

In this matched-subject-designed study, the subjects were randomly recruited using a double-blind, placebo-controlled parallel-groups, fixed-dose, and pretest–posttest design. Participants were divided in two groups receiving wither crocodile blood (CB; *n* = 8) or placebo treatment (PL; *n* = 8) for 18 days. The participants in this study were assigned to either the CB group or the placebo group. The participants in each group were matched for body mass, BMI, and body fat percentage. On the first visit to the laboratory, each participants’ body mass (kg) (Omron^R^ Karada Scan, model HBF362, Shinagawa, Tokyo, Japan) and height (m) were measured, and BMI (kg/m^2^) was calculated. Systolic and diastolic blood pressure was recorded using a digital automated blood pressure monitor (model SEM-1, Omron^R^, Shinagawa, Tokyo, Japan).

**Figure 1:**
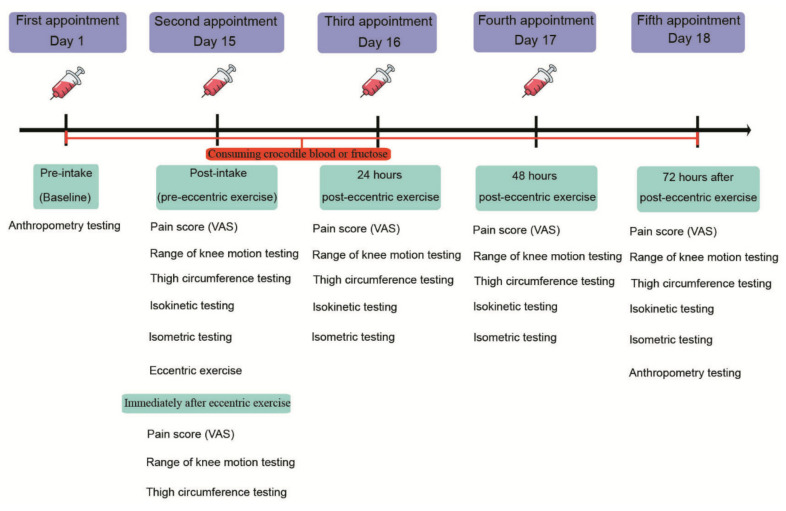
Experimental design.

Prior to the commencement of the eccentric exercise protocol, participants consumed 4 capsules of CB (crocodile blood powder: 250 mg/ capsule; Sriracha Moda Co., Ltd., Thailand) or placebo (fructose: 250 mg/capsule; Sriracha Moda Co., Ltd., Thailand) every day (twice a day: after breakfast (at 8.00 a.m.) and after dinner (at 6.00 p.m.)) for 18 days. The CB and placebo capsules were the same color to ensure that the participants and researchers were blinded to the type of capsule consumed. Eleven days after the initial visit to the laboratory, a 24 h dietary recall via structured interview was performed, with participants asked to recall all food and drinks consumed in the previous 24 h. This dietary recall was repeated over the following 7-day period (days 11 to 17), and each participants’ average calorie intake was calculated. On the second visit to the laboratory (day 15), the participants were asked to complete an eccentric exercise protocol on an isokinetic dynamometer. Visual analogue pain scores (VAS), range of knee motion (ROM), thigh circumference, maximal voluntary contraction (MVC) and maximal voluntary isometric contraction (MVIC) strength, anthropometric measures (body mass and BMI), and blood inflammatory and biochemical markers were evaluated at pre-determined time-points (Figure 1).

### 2.4. Procedures

#### 2.4.1. Eccentric Exercise Protocol

The eccentric exercise protocol was carried out using an isokinetic dynamometer (model Biodex System 4 Pro, Biodex^R^, Shirley, New York, NY, USA). Participants sat reclined at an angle of 85° on the dynamometer chair with the lateral condyle of the knee aligned with the center of the attachment arm. The participants were strapped securely to the chair at the thigh, waist, and across the shoulders to limit any extraneous movement. The participants’ chair position settings were recorded to enable the same position to be used across repeated measurements. The participants initially performed an eccentric warm-up on the dominant leg, consisting of 2 sets of 10 repetitions at an intensity of 50% of concentric one-repetition maximum (1RM) [4]. The participants were then asked to complete an eccentric exercise protocol consisting of 7 sets of 10 MVCs (15 s rest between contractions) at an intensity of 150% of concentric 1RM and at an angular velocity of 60°s^−1^. A 3 min rest period was provided between sets. All participants wore shorts and a t-shirt throughout the study.

#### 2.4.2. Muscle Strength Assessment

All participants performed a standardized 10 min warm-up on a cycle ergometer (load weight = 1 kg at 60 revolutions per min (RPM). The participants then completed 2 sets of 5 MVCs separated by 3 min of rest on the isokinetic dynamometer at an angular velocity of 60° s^−1^, and peak torque was recorded. After a 3 min rest period, the participants completed the MVIC protocol (at an angle of 60°) consisting of 2 sets of 5 contractions (15 s rest between contractions) with a 3 min rest interval between sets. The MVC and MVIC tests were completed at pre-eccentric exercise, 24 h, 48 h, and 72 h.

#### 2.4.3. Blood Collection and Biochemical Measures

A 15 mL blood sample was drawn from each participant’s median cubital vein after overnight fasting. Fasting blood glucose was measured in plasma using NaF as an anticoagulant. BUN, AST, ALT, ALK, LDH, ALB, CK, IL-6, and TNF-α serum levels were measured. CBC testing was performed in whole blood using EDTA as an anticoagulant. 

The blood samples (15 mL) were centrifuged at 5000 rpm for 10 min to obtain either plasma or serum. LDH, CK, glucose, blood urea nitrogen (BUN), aspartate transaminase (AST), alanine transaminase (ALT), alkaline phosphatase (ALK), complete blood count (CBC), and albumin (ALB) were analyzed. Serum interleukin-6 (IL-6) and tumor necrosis factor alpha (TNF-α) concentrations were determined using a human IL-6 ELISA Kit (Lot number: RAB0306, Sigma-Aldrich^TM^, Saint Louis, USA) and a human tumor necrosis factor alpha ELISA Kit (Lot number: RAB0476, Sigma-Aldrich^TM^, Saint Louis, MO, USA), respectively. The inflammatory cytokines were then measured using a microplate reader (model Sunrise, TECAN^R^, Männedorf, Switzerland). The blood samples were sent to the Community Health Care service, Faculty of Medical Technology, Mahidol University, Thailand. LDH, CK, glucose, blood urea nitrogen, aspartate transaminase, alanine transaminase, alkaline phosphatase, and albumin concentrations were analyzed by an automatic biochemistry analyzer (Cobas c 501, Hoffmann-La Roche Ltd., Rotkreuz, Switzerland).

Blood samples were drawn at baseline, post-intake of CB or PL, 24 h, and 48 h after eccentric exercise (Figure 1).

#### 2.4.4. Thigh Circumference

The participants’ dominant leg was assessed for muscle inflammation and swelling by measuring mid-thigh circumference. The mid-point of each participant’s thigh was initially identified (and marked) by measuring the halfway point between the greater trochanter and the lateral epicondyle of the femur. As previously described by [1], the mid-thigh circumference was assessed using a measuring tape (model CWD-0422, Windy^R^, Bangkok, Thailand) with the average value of 3 measures recorded. Thigh circumference measurements were taken pre-exercise and post-exercise, 24 h, 48 h, and 72 h after eccentric exercise (Figure 1).

#### 2.4.5. Range of Knee Motion

Range of knee motion (ROM) was measured by asking the participants to voluntarily flex the knee of their dominant leg whilst laying in a prone position on a portable medical bed. As previously described [1], the knee joint angle of each participants’ dominant leg was measured, using a goniometer (Model 47, Prestige^R^ medical goniometer, Taiwan), at universal landmarks (greater trochanter, lateral malleolus, and lateral epicondyle of the femur). The measurements were taken immediately pre- and post-exercise, and 24 h, 48 h, and 72 h after eccentric exercise (Figure 1). 

#### 2.4.6. Pain Score Measurement

Muscle pain was assessed using a 100 mm VAS, labeled “no pain” and “unbearable pain” at opposite ends [16]. Participants were requested to rate the sensation of discomfort in the quadriceps of their dominant leg before the exercise, immediately after the exercise, and at 24 h, 48 h, and 72 h after the exercise.

### 2.5. Statistical Analysis

The PASW Statistics for Windows, Version 18.0 (SPSS Inc., Chicago, IL, USA) was used for all statistical analysis. A Shapiro–Wilk test was used to check the normality of the data, and Levene’s test was used to check for homogeneity of variance between treatments. For VAS, knee ROM, and thigh circumference parameters, the data were converted to change (Δ) from pre-eccentric exercise values. Two-way repeated measures analysis of variance (ANOVA) was used to evaluate the main effects and interaction effects pre-exercise, post-exercise, and 24 h, 48 h and 72 h after the exercise. Furthermore, a post hoc Fisher’s Least Significant Difference (LSD) adjustment was applied for pairwise comparisons with significant interaction effects. The biochemical data were analyzed using a two-tailed *t*-test to compare the crocodile blood supplementation group and the placebo group. A *p*-value of less than 0.05 was considered statistically significant. The biochemical data were analyzed using a two-tailed t-test to compare the crocodile blood supplementation group with the placebo group. A *p*-value of less than 0.05 was considered statistically significant. The means ± 95% confidence intervals (95% CI) are presented for all recovery parameters. The estimated marginal means (and 95% CIs) are reported for the biochemical data.

### 2.6. Power Calculation

The sample size was estimated from a sample calculation (G*Power) with an alpha level of 0.05, a power (1-β) of 0.80, and a medium effect size of 0.5 and suggested that a total *n* = 12 would be sufficient.

## 3. Results

The participants’ anthropometric data and dietary intake were similar between groups after 15 days of supplementation (*p* > 0.05; Table 1).

### 3.1. Effects on Maximum Voluntary Isometric Contraction (MVIC)

**Figure 2:**
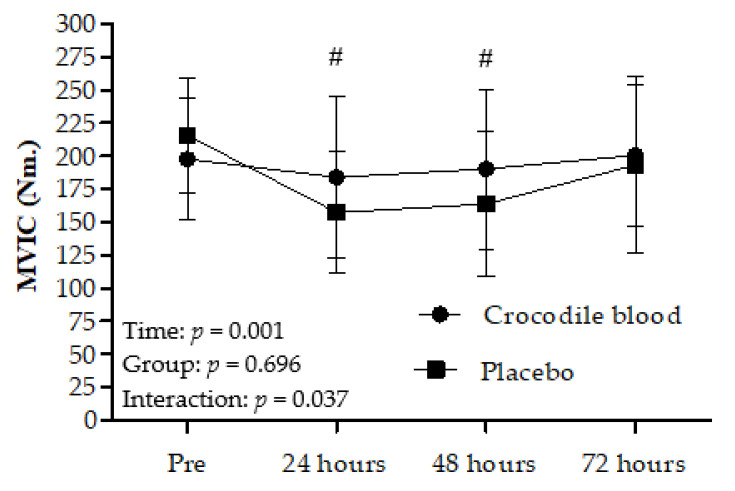
Maximum voluntary isometric contraction (MVIC) peak torque before eccentric exercise (Pre) and 24 h, 48 h, and 72 h after eccentric exercise (mean ± 95% CI). Data were analyzed using a two-way repeated-measures ANOVA with post hoc Fisher’s Least Significant Difference (LSD), in comparison to values before eccentric exercise. # = MVIC significantly different in the placebo group compared to pre-eccentric exercise values (*p* < 0.001).

For the MVIC peak torque, there was no significant difference between the groups; there was a significant difference in time effects (*p* = 0.001) and interaction (group*time) effects (*p* = 0.037). Moreover, there was no significant difference between times (*p* > 0.05) in the CB group. In contrast, the MVIC peak torque in the PL group showed a significant difference, with a greater decrease at 24 h (*p* = 0.001) and 48 h (*p* = 0.001) compared to the value before the eccentric exercise. The MVIC peak torque remained unchanged with respect to pre-eccentric exercise values throughout the recovery period in the CB group (*p* > 0.05; Figure 2).

### 3.2. Effects on Maximum Voluntary Isokinetic Contraction (MVC)

**Figure 3:**
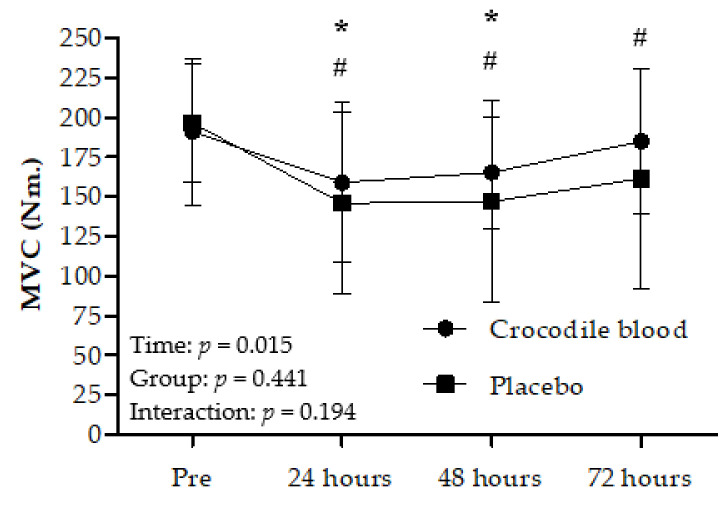
Maximum voluntary contraction (MVC) peak torque before eccentric exercise (Pre) and 24 h, 48 h, and 72 h after eccentric exercise. (mean ± 95% CI). Data were analyzed using a two-way repeated-measures ANOVA with post hoc Fisher’s Least Significant Difference (LSD) in comparison to pre-eccentric exercise values. # = Significant difference in the placebo group in comparison to pre-eccentric exercise values (*p* < 0.05) * = Significant difference in the crocodile blood group in comparison to pre-eccentric exercise values (*p* < 0.05).

For MVC peak torque, there was no significant difference between groups (*p* > 0.05) and for interaction effects (*p* > 0.05). A significant time effect (*p =* 0.015) was noted, with a greater decrease in MVC values in the CB group at 24 h (*p* = 0.021) and 48 h (*p* = 0.012) after exercise compared to pre-eccentric exercise values. The MVC peak torque significantly changed from pre-eccentric exercise values 24 h and 48 h after exercise in both groups. However, the MVC peak torque significantly changed in the PL group throughout the recovery period at 24 h (*p* = 0.003), 48 h (*p* = 0.008), and 72 h (*p* = 0.044) compared to pre-eccentric exercise values (Figure 3).

### 3.3. Effects on Visual Analog Scale (VAS) Scores 

**Figure 4:**
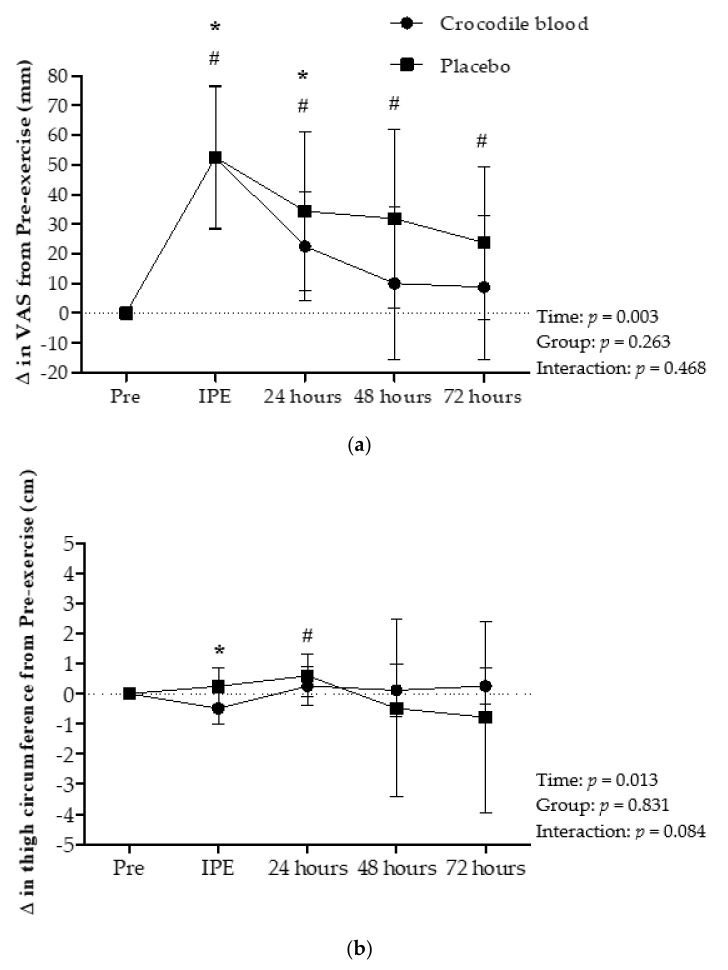

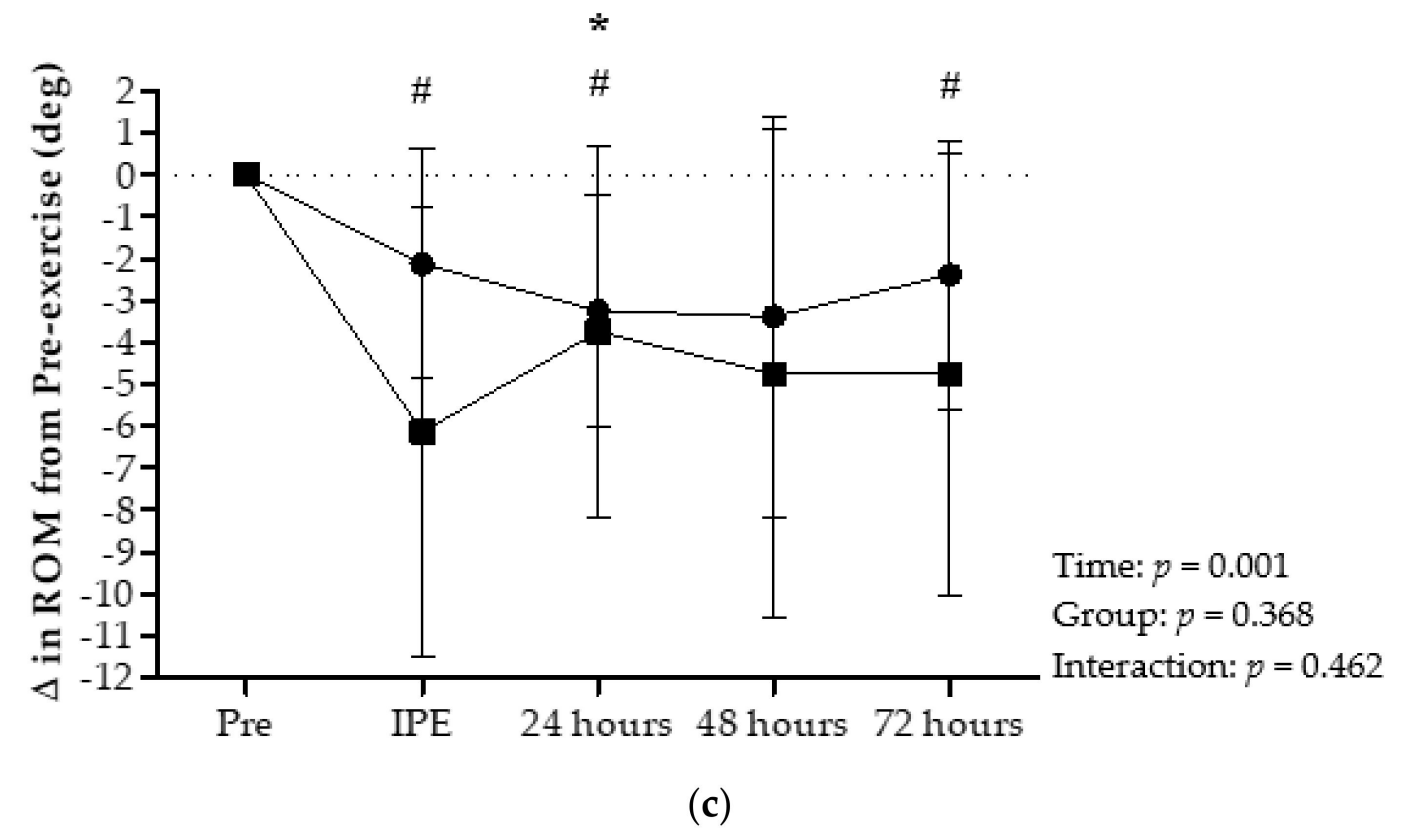
(**a**) Changes in visual analogue scale (VAS) scores for pain with respect to pre-eccentric exercise (Pre) values, (**b**) thigh circumference, and (**c**) range of knee motion (ROM), immediately after eccentric exercise (IPE) and 24 h, 48 h, and 72 h after eccentric exercise (mean ± 95% CI). Data were analyzed using a two-way repeated-measures ANOVA with post hoc Fisher’s Least Significant Difference (LSD) in comparison to from pre-eccentric exercise values. # = Significant difference in the placebo group in comparison to pre-eccentric exercise values (*p* < 0.05), * = Significant difference in the crocodile blood group in comparison to pre-eccentric exercise values (*p* < 0.05).

VAS pain scores showed no significant difference between groups (*p* = 0.263) and for interaction effects (*p* = 0.468). VAS pain scores differences were significant for time effects (*p* = 0.003). VAS pain scores in the CB group were generally higher immediately post-eccentric exercise (*p* < 0.001) and 24 h (*p* = 0.010) post-eccentric exercise compared to pre-eccentric exercise scores. In addition, VAS pain scores in the PL group were significantly higher throughout the recovery period at immediately (*p* < 0.001), 24 h (*p* = 0.009), 48 h (*p* = 0.020), and 72 h (*p* = 0.035) post-eccentric exercise compared to pre-eccentric exercise (Figure 4a). 

### 3.4. Effects on Thigh Circumference

The thigh circumference showed a significant time effect (*p* = 0.013). In addition, CB was found to generally decrease thigh circumference only immediately after eccentric exercise (*p* = 0.031) in comparison with pre-eccentric exercise. In the PL group, thigh circumference was increased at 24 h (*p* = 0.047) after eccentric exercise compared to pre-eccentric exercise (Figure 4b). In addition, there was no significant difference between groups (*p* = 0.831) and interaction effects (*p* = 0.084).

### 3.5. Effects on Range of Knee Motion (ROM)

For knee ROM, there was no significant difference between groups (*p* = 0.368) and interaction effects (*p* = 0.462). However, knee ROM showed a significant time effect (*p* = 0.001). Knee ROM in the CB group showed a significant difference 24 h (*p* = 0.013) post-eccentric exercise compared to pre-eccentric exercise values. Also, Knee ROM in the PL group showed a dramatical difference immediately (*p* = 0.015), 24 h (*p* = 0.048), and 72 h (*p* = 0.038) after eccentric exercise (Figure 4c).

### 3.6. Effects on Serum Cytokines and Enzymes

The analysis of serum cytokine and enzyme data and of inflammatory markers, except for creatine kinase, showed no difference in the main effect between groups, times, and interaction effects (group*time) for any parameter during the 48 h post-eccentric exercise recovery period (*p* > 0.05; Table 2). Similarly, after 15 days of supplementation (post-intake), there were no significant differences in any biochemical parameter between treatments (*p* > 0.05; Table 3). 

### 3.7. Effects on Creatine Kinase (CK)

**Figure 5:**
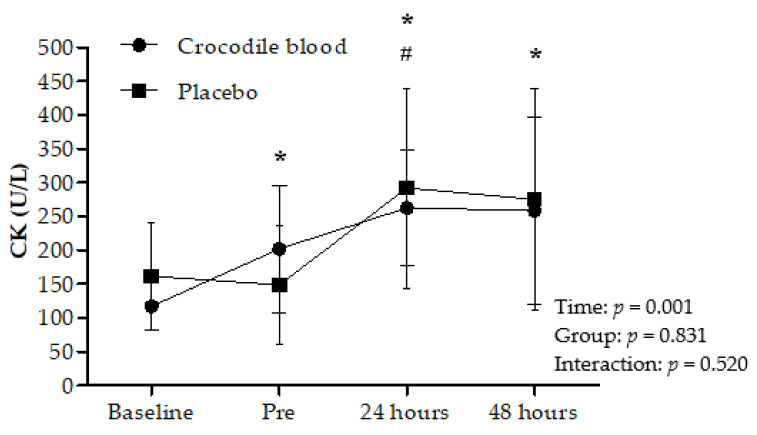
The absolute values of creatine kinase (CK) at baseline (before supplementation intake), before eccentric exercise (Pre) and 24 h and 48 h after supplementation intake (mean ± 95% CI). Data were analyzed using a two-way repeated-measures ANOVA with post hoc Fisher’s Least Significant Difference (LSD) in comparison to from baseline values. # = Significant difference in the placebo group compared to baseline values (*p* < 0.05) * = Significant difference in the crocodile blood group compared to baseline values (*p* < 0.05).

The absolute values of creatine kinase showed no significant difference between groups (*p* = 0.831) and interaction effects (*p* = 0.520). However, CK showed a significant time effect (*p <* 0.001). CK level in the CB group was significantly higher before eccentric exercise (*p* = 0.024) and 24 h (*p* = 0.001) and 48 h (*p* = 0.013) after eccentric exercise compared to baseline values (pre-intake CB supplementation). Moreover, CK level in the PL group was significantly higher 24 h (*p* = 0.048) after eccentric exercise compared to baseline values (pre-intake placebo) (Figure 5).

## 4. Discussion

The main finding of this study is that CB supplementation showed a preventative effect on markers of muscle damage including MVIC and thigh circumference compared to a placebo treatment after eccentric exercise. Thigh circumference showed no significant difference between groups and interaction effects, but significant difference in time effect. In the CB group, thigh circumference decreased only immediately after eccentric exercise, indicating that CB helped reduce muscle swelling at that specific time. On the other hand, thigh circumference increased in the PL group 24 h after eccentric exercise compared to its measure before eccentric exercise, indicating increase muscle swelling. Therefore, the results indicated that CB supplementation aids in reducing muscle swelling immediately after eccentric exercise. CB supplementation showed no interaction effect on MVC, VAS, ROM, IL-6, TNF-α, LDH, and CK. The biochemical assessment of CB supplementation provided evidence that an acute 1 g day^−1^ of CB supplementation should be safe for human consumption. Several research studies support the hypothesis that CB supplementation is safe in rats and shows no acute and chronic toxicity [7]. Moreover, rats were administered a 50% lethal dose (LC_50_) corresponding to more than 5000 mg per 1 kg of body weight [7].

Studies reported that CB supplementation has anti-inflammatory properties [7,10,13], CB supplementation may impact on recovery compared with the placebo treatment, suggesting that CB may affect DOMS. Our findings challenge the notion that CB can benefit the recovery from eccentric or unaccustomed exercise, whilst our findings are similar to those of other works, which studied saffron and taurine supplementation and documented a preventative effect [1,17]. 

In the present study, ethical considerations prevented the selection of a higher CB dosage. CB did not positively impact the levels of the inflammatory mediators IL-6 and TNF-α after eccentric exercise, and a similar trend was observed for cytokine levels after delayed-onset muscle soreness in studies on fish oil and isoflavones [18]. Increased serum CK and LDH levels are used as indicators of inflammation as a consequence of DOMS. Creatine kinase levels in the CB group were significantly increased after 24 h and 48 h of eccentric exercise, whereas LDH levels were not. However, CB supplementation did not reduce CK elevation after eccentric exercise, which is different from the reduction effect of saffron and taurine on CK elevation [1,17]. Muscle cell-released IL-6 increased and peaked at approximately 2–4 h after eccentric exercise, whereas TNF-α did not [2,19]. In contrast, TNF-α increased and peaked 48 h after eccentric exercise [17]. In our study, however, IL-6 and TNF-α were not induced by eccentric exercise, which is different from what observed on previous reports [2,19]. This could be due to different detection times for cytokines and different eccentric protocols. This observation further supports the inference that the dose of crocodile blood supplementation in this study (1 g day^−1^ over 18 days) may affect the maintenance of MVIC peak torque. Blood crocodile from *Crocodylus Siamensis* contains hemoglobin and fragmented hemoglobin which have been demonstrated to have both antibacterial and antioxidant properties. The digested hemoglobin products, which include peptides and 20 amino acids, might have helped maintain MVIC peak torque in the CB group after eccentric exercise. The amino acids found in *C. siamensis* hemoglobin were Val, Leu, Pro, His, Tyr, Trp, Phe, Cys, Glu, Asp, Lys, and Arg. The antioxidant activity of peptides containing His and Tyr might involve the ability to chelate reactive metal species and the lipid peroxide radical-catching capacity due to the imidazole ring and aromatic ring, respectively [9,10].

Moreover, in light of our biochemical data showing no adverse outcomes of the 1 g day^−1^ dose of CB over the 15-day supplementation period, it is recommended that future studies attempt to examine the efficacy of higher CB doses to promote recovery from exercise-induced muscle damage. Our current findings indicate that crocodile blood does not contain harmful components and contains proteins, minerals (iron, calcium, sodium, phosphate, and magnesium), and vitamins (A and B_1_, B_2_, B_6_, and B_12_) [7]. To our knowledge, this is the first report showing that 1.0 g per day of crocodile blood supplementation should be safe for human consumption, as confirmed by the stable values of biochemical parameters after its administration (Table 3).

Our findings challenge the notion that CB can helps the recovery from eccentric or unaccustomed exercise. Our findings are in line with previous studies that investigated the effects of dietary fish oil and isoflavones supplementation on DOMS and reported little benefit [18] In contrast, saffron and taurine supplementation was shown to exert a preventative effect on DOMS through an anti-inflammatory effect [1,17].

There are several limitations in our study, including the small sample size, impacting on the power to detect differences between treatments, and the potentially low dose and short loading duration of CB supplementation. In our study, the CB dose could not be varied due to ethical restrictions that prevent the CB consumption above the maximum allowance level (1 g per day) recommended by the Thailand Food and Drug administration [7]. Therefore, future studies should address these limitations to comprehensively determine whether CB is useful as an ergogenic aid.

## 5. Conclusions

An 18-day supplementation of 1 g day^−1^ of CB helps to maintain peak muscle force or DOMS compared to a placebo after eccentric exercise. In addition, biochemical analysis provided evidence that 1 g day^−1^ of CB supplementation should be safe for human consumption.

## Figures and Tables

**Table 1:** Between-treatment comparisons of anthropometric and dietary intake data after 15 days of supplementation (mean ± SD).

	Placebo (*n* = 8)	CB (*n* = 8)	^†^*p*-Value
Mass (kg)	65.73 ± 7.37	64.72 ± 5.79	0.764
BMI (kg/m^2^)	22.09 ± 1.7	21.4 ± 2.13	0.457
Body fat (%)	18.69 ± 4.6	14.2 ± 5.7	0.102
Thigh circumference (cm)	53.2 ± 3.05	53.1 ± 3.4	0.936
Average kcal Intake (7-days)	1411 ± 200	1562 ± 406	0.360
Baseline 1-RM (Nm)	196.59 ± 37.40	190.85 ± 46.56	0.790

1-RM = 1-repetition maximum ^†^ *p*-value was calculated by the independent *t*-test.

**Table 2:** Comparison of serum cytokines and enzymes between the CB supplementation and the placebo treatments (mean ± 95% CI).

	Baseline	Post-Intake (Pre-Eccentric Exercise)	24 h	48 h	^‡^*p*-Value		
					Main Effect		Interaction Effect
					Group	Time	Group * Time
IL-6 (pg/mL)							
CB (*n* = 8)	12.58 (−1.77, 26.94)	15.47 (−12.34, 43.28)	20.91 (−19.03, 60.86)	24.88 (−6.13, 55.89)	0.525	0.853	0.676
Placebo (*n* = 8)	28.28 (−11.61, 68.17)	43.68 (−36.09, 123.45)	17.96 (2.30, 33.61)	13.48 (−1.17, 28.12)			
TNF-α (pg/mL)							
CB (*n* = 8)	60.37 (19.64, 101.11)	47.38 (7.45, 87.30)	65.88 (−7.77, 139.52)	70.00 (7.97, 132.03)	0.362	0.857	0.958
Placebo (*n* = 8)	105.50 (19.46, 191.54)	93.75 (4.59, 182.91)	99.00 (18.48, 179.52)	101.25 (8.76, 193.74)			
LDH (U/L)							
CB (*n* = 8)	275.38 (237.09, 313.66)	305.50 (258.65, 352.35)	304.50 (261.93, 347.07)	308.38 (261.35, 355.40)	0.221	0.167	0.444
Placebo (*n* = 8)	268.00 (241.61, 294.39)	257.00 (181.97, 332.03)	324.88 (246.14, 403.61)	270.38 (244.00, 296.75)			

^‡^*p*-value was calculated by two-way repeated-measures ANOVA.

**Table 3:** Comparison of biochemical parameters between the CB supplementation and the placebo treatments after 15 days supplementation (estimated marginal means ± 95% CI).

	Baseline		^†^*p*-Value	Post-Intake (Pre-Eccentric Exercise)		^†^*p*-Value
	CB (*n* = 8)	Placebo (*n* = 8)		CB (*n* = 8)	Placebo (*n* = 8)	
Fasting blood glucose (mg/dL)	87.4 (80.1, 94.7)	85.0 (80.02, 89.98)	0.536	83.5 (78.0, 89.0)	86.6 (80.3, 93.0)	0.393
Hemoglobin (g/dL)	15.5 (15.1, 15.9)	14.3 (13.0, 15.6)	0.050	15.2 (14.6, 15.7)	14.2 (13.1, 15.2)	0.063
Hematocrit (%)	46.2 (45.1, 47.2)	42.7 (40.3, 45.0)	0.006 *	45.4 (44.2, 46.5)	42.6 (39.6, 45.5)	0.057
Red cell count (×10^12^/L)	5.2 (5.0, 5.4)	5.5 (4.8, 6.1)	0.380	5.1 (5.0, 5.3)	5.5 (4.8, 6.2)	0.204
White blood cell count (×10^9^/L)	5.9 (4.9, 6.8)	7.7 (5.9, 9.6)	0.052	5.8 (5.0, 6.6)	7.4 (5.5, 9.4)	0.083
Neutrophils (% WBC)	48.2 (42.4, 53.9)	51.6 (45.2, 58.1)	0.359	49.6 (42.8, 56.4)	53.0 (46.0, 60.0)	0.429
Lymphocyte (% WBC)	40.4 (35.7, 45.1)	38.0 (31.6, 44.4)	0.486	39.3 (33.2, 45.5)	36.0 (30.2, 41.8)	0.364
Monocyte (% WBC)	8.0 (5.8, 10.2)	6.5 (5.7, 7.3)	0.146	7.9 (6.8, 9.0)	7.1 (6.5, 7.6)	0.118
Eosinophil (% WBC)	2.9 (1.5, 4.3)	3.4 (2.3, 4.5)	0.546	2.6 (0.5, 4.7)	3.3 (2.1, 4.4)	0.528
Basophils (% WBC)	0.5 (0.3, 0.6)	0.5 (0.3, 0.7)	1.000	0.6 (0.3, 0.8)	0.4 (0.2, 0.6)	0.251
Platelet count (x10^9^/L)	231.8 (180.8, 282.7)	272.6 (239.1, 306.2)	0.136	237.6 (205.8, 269.4)	287.6 (232.0, 343.3)	0.086
Alkaline phosphatase (U/L)	77.0 (62.9, 91.1)	70.9 (58.5, 83.2)	0.453	74.3 (61.2, 87.3)	69.6 (56.8, 82.4)	0.560
Aspartate transaminase (U/L)	19.8 (15.6, 23.9)	15.9 (14.2, 17.5)	0.059	19.8 (17.0, 22.5)	16.6 (12.6, 20.7)	0.157
Alanine transaminase (U/L)	19.3 (9.9, 28.6)	16.5 (12.6, 20.4)	0.531	17.6 (10.9, 24.3)	17.3 (10.1, 24.4)	0.929
Blood urea nitrogen (mg/dL)	15.2 (12.7, 17.6)	14.7 (12.7, 16.7)	0.728	14.3 (11.7, 16.8)	13.3 (10.9, 15.7)	0.517
Albumin (gm/dL)	4.6 (4.4, 4.7)	4.7 (4.5, 4.9)	0.419	4.6 (4.5, 4.7)	4.6 (4.4, 4.9)	0.910

^†^*p*-value was calculated by the independent *t*-test. * = significant difference between baseline and post-intake values calculates by the independent t-test (*p* < 0.05).

## Data Availability

The data presented in this study are available on request from the corresponding author, due to privacy restrictions.
